# Attentional sampling of visual and auditory objects is captured by theta-modulated neural activity

**DOI:** 10.1111/ejn.15514

**Published:** 2021-11-10

**Authors:** Michael Plöchl, Ian Fiebelkorn, Sabine Kastner, Jonas Obleser

**Affiliations:** 1 Department of Psychology, University of Lübeck, Lübeck, Germany; 2 Princeton Neuroscience Institute, Princeton University, Princeton, New Jersey, USA; 3 Department of Psychology, Princeton University, Princeton, New Jersey, USA; 4 Center of Brain, Behavior, and Metabolism, University of Lübeck, Lübeck, Germany

**Keywords:** alpha, attention, auditory, cross-modal, EEG, phase opposition, rhythm, sampling, theta, visual

## Abstract

Recent evidence suggests that visual attention alternately samples two behaviourally relevant objects at approximately 4 Hz, rhythmically shifting between the objects. Whether similar attentional rhythms exist in other sensory modalities, however, is not yet clear. We therefore adapted and extended an established paradigm to investigate visual and potential auditory attentional rhythms, as well as possible interactions, on both a behavioural (detection performance, *N* = 33) and a neural level (EEG, *N* = 18). The results during unimodal attention demonstrate that both visual- and auditory-target detection fluctuate at frequencies of approximately 4–8 Hz, confirming that attentional rhythms are not specific to visual processing. The EEG recordings provided evidence of oscillatory activity that underlies these behavioural effects. At right and left occipital EEG electrodes, we detected counter-phasic theta-band activity (4–8 Hz), mirroring behavioural evidence of alternating sampling between the objects presented right and left of central fixation, respectively. Similarly, alpha-band activity as a signature of relatively suppressed sensory encoding showed a theta-rhythmic, counter-phasic change in power. Moreover, these theta-rhythmic changes in alpha power were predictive of behavioural performance in both sensory modalities. Overall, the present findings provide a new perspective on the multimodal rhythmicity of attention.

## 1 | INTRODUCTION

Rhythmic behavioural patterns, especially the ones serving active exploration of the environment (e.g., eye movements) or communication (e.g., speech production), typically occur at frequencies in the theta range, that is, at frequencies between around 3–8 Hz (Benedetto & Morrone, 2019; Fiebelkorn & Kastner, 2019; Otero-Millan et al., 2008; Poeppel & Assaneo, 2020; Schroeder et al., 2010). Theta oscillations also seem to play a prominent role in the internal processing of the external environment (i.e., attention-related sampling). Examples include neural responses to sensory stimuli (e.g., visual lambda responses; Dimigen et al., 2009), stimulus detection (Fiebelkorn et al., 2013, 2018; Landau & Fries, 2012), spatial navigation (O’Keefe & Recce, 1993), working memory (Jensen & Tesche, 2002) and neural entrainment to external rhythms (Henry et al., 2014). These observations have led to the notion that perception and action may be coordinated via a common theta rhythm (Benedetto et al., 2020; Fiebelkorn & Kastner, 2019; Schroeder et al., 2010; Schroeder & Lakatos, 2009).

The same network of cortical and subcortical structures directs both the sensory (i.e., attention-related boosts in sensory processing) and the motor (e.g., saccadic eye movements) functions associated with environmental sampling (Fiebelkorn & Kastner, 2020). Theta rhythms in this large-scale ‘attention network’ may help to temporally organize neural activity associated with perception and action, helping to prevent potential functional conflicts (Fiebelkorn & Kastner, 2019). With regard to perception, a number of studies have provided evidence that visual attention samples space or objects rhythmically and in sequence with sampling occurring at a frequency in the theta range (i.e., 3–8 Hz; Busch & VanRullen, 2010; Fiebelkorn et al., 2013; Fiebelkorn et al., 2018; Helfrich et al., 2018; Gaillard et al., 2020; Landau & Fries, 2012; Landau et al., 2015). More specifically, some studies have suggested that single objects (or locations) are sampled at a rate of ~8 Hz, while two simultaneously attended objects are alternately sampled at a rate of ~4 Hz (Fiebelkorn et al., 2013; Holcombe & Chen, 2013; Landau et al., 2015; Landau & Fries, 2012; Re et al., 2019; Senoussi et al., 2019; VanRullen, 2016b). That is, these studies have proposed that there is a constant sampling frequency (~8 Hz) that can be split across multiple objects or locations. Recent evidence further suggests that periods of attention-related sampling (i.e., associated with enhanced visual-target detection) rhythmically alternate with periods characterized by a higher likelihood of attentional shifting, with theta rhythms serving as the clocking mechanism for these alternating sensory- and motor-related attentional states (Fiebelkorn & Kastner, 2019; Hogendoorn, 2016; Ronconi et al., 2017; Senoussi et al., 2019).

If one function of theta rhythms in the attention network is to temporally coordinate sensory and motor functions, then attentional rhythms might not be restricted to the visual modality. That is, similar coordination might be necessary when sampling is occurring within a different sensory modality (i.e., the auditory modality) or across multiple sensory modalities. In line with this idea, evidence suggests that attention is supramodal (Green et al., 2011; Shomstein & Yantis, 2004; Wang et al., 2016). Lakatos et al. (2009), for example, demonstrated that an attention-related phase reset in one modality also resets the phase of theta oscillations in the primary cortical areas of other modalities. Several studies have now specifically investigated whether attentional rhythms influence the auditory modality. However, while some of the findings provide evidence for rhythmic fluctuations of auditory attention (Ho et al., 2017; Kayser, 2019; Ng et al., 2012) other studies argue against their existence (Ilhan & VanRullen, 2012; Zoefel & Heil, 2013). A likely reason for these conflicting results and conclusions is that the experimental paradigms and methods varied widely across the studies and were rarely comparable to those used in visual experiments. Here, we examine whether attentional rhythms influence the auditory modality using an experimental paradigm that closely mirrors the design of studies that have demonstrated attentional rhythms during visual processing. That is, we investigated attentional rhythms in behavioural performance during an established visual paradigm augmented with a congruent auditory task, allowing us to examine whether similar oscillatory mechanisms are present in both modalities. Moreover, by testing detection performance during both unimodal attention and bimodal attention, we address the question of whether visual attention and auditory attention are governed by a common underlying theta rhythm. For example, differences in frequency between visual and auditory attentional rhythms would suggest that attention in the two modalities is governed by different underlying processes. By contrast if the frequencies were similar in the unimodal conditions and then dropped to half the rate in the bimodal condition (e.g., from 4 to 2 Hz) this would serve as an indicator that attention is a supramodal mechanism that is equally split across the number of presented objects (which increases from 2 to 4). Finally, there is the possibility that the frequency of attentional rhythms stays the same across modalities and attention conditions. This in turn might be explained by a single supramodal attentional spotlight, which samples different objects at a rate of 4 Hz but not in a particular sequence.

Moreover, rhythmic patterns in behavioural data are also reflected in theta or theta-modulated neural activity. Recently, it has been shown that theta-band activity in the attention network predicts detection performance (Castellano et al., 2014; Fiebelkorn et al., 2018, 2019; Helfrich & Knight, 2016; Helfrich et al., 2018) and that attention-related theta-band activity also modulates higher frequency bands that are commonly linked to attention, such as gamma (>30 Hz; Fiebelkorn et al., 2018; Landau et al., 2015) and in particular alpha bands (8–13 Hz; Fiebelkorn et al., 2019; Jia et al., 2017; Song et al., 2014; VanRullen, 2016b). Here, we recorded EEG data during task performance to investigate whether theta-band activity differentially modulates modality-specific alpha-band activity, which has been repeatedly associated with the suppression of task-irrelevant sensory information (Foxe & Snyder, 2011).

## 2 | METHODS

### 2.1 | Participants

We recorded detection performance of 41 healthy participants, including 29 participants from whom we simultaneously obtained EEG data. We excluded those participants from further analyses whose performance (detection rate, false alarms, and eye movements during the task) deviated more than 1.96 standard deviations from the group mean in at least one of the conditions (*n* = 8, see also Section 2.2). We had to exclude another four subjects from EEG analysis only, because of poor data quality or technical problems during the recording. This left us with a total of 33 behavioural (14 male, 19 female; age range: 20–33 years) and 18 EEG data sets for further analyses. All participants gave written informed consent and received financial compensation or course credit. All experimental procedures were conducted in accordance with the Declaration of Helsinki and approved by the ethics review board of the University of Lübeck.

### 2.2 | Experimental Procedures

In order to investigate attentional rhythms in the visual and auditory domain, we adopted and expanded an established target detection paradigm (Landau & Fries, 2012). Prior to the measurements, participants were instructed about the experimental task and procedure. Over the course of the whole experiment, each participant performed four separate experimental blocks: one block comprising only visual targets (unimodal visual attention condition), one block comprising only auditory targets (unimodal auditory attention condition), and two blocks during which visual and auditory target stimuli were randomly interleaved (bimodal attention condition). Every participant started with a bimodal block. The subsequent block order was quasi-randomized. Each block consisted of 220 experimental trials and 44 catch trials (see below).

**FIGURE 1:**
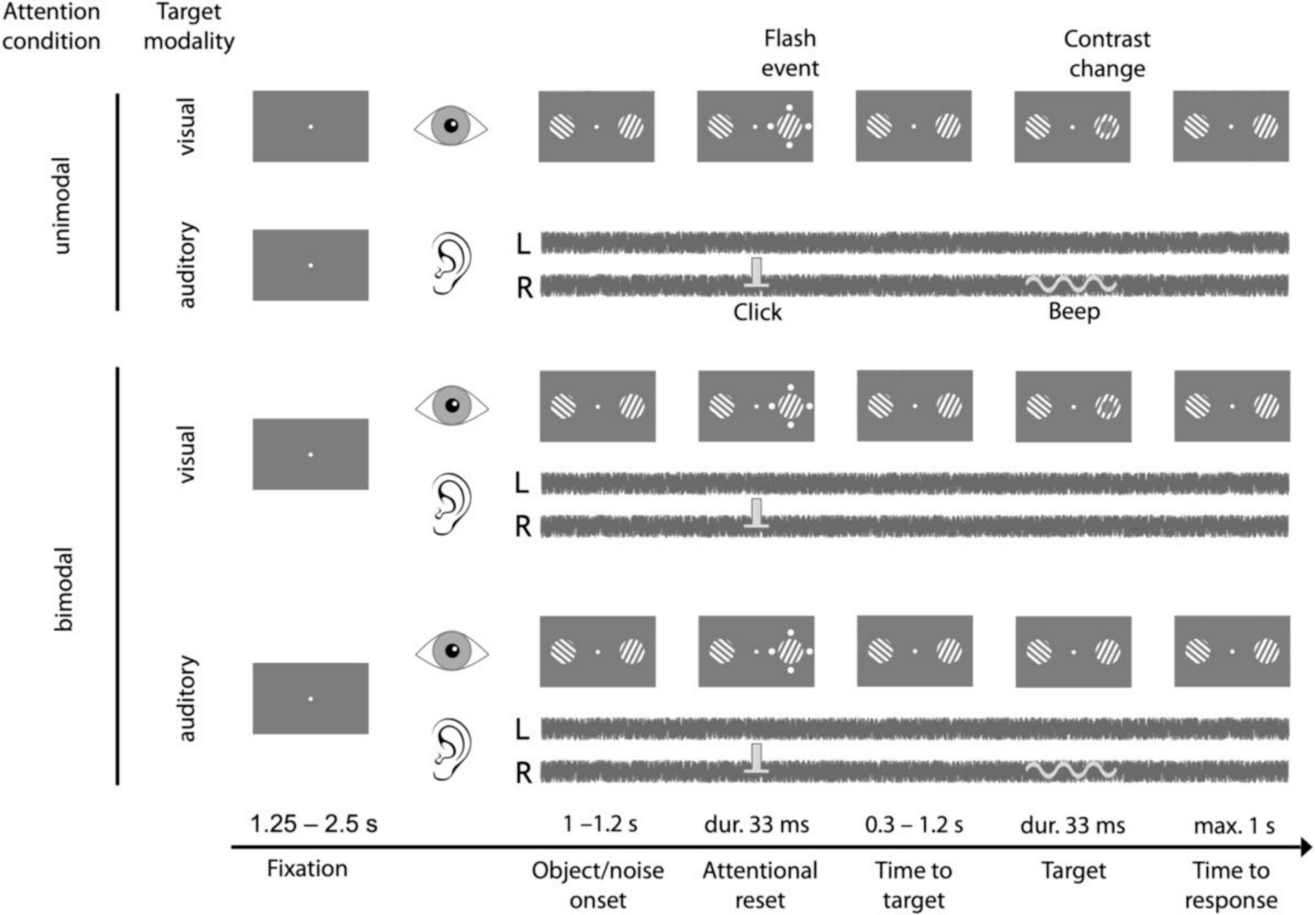
Audio-visual detection task. In the unimodal visual-attention condition (top row), participants fixated the centre of the screen and two objects appeared. Attention was reset towards one of the objects using a brief attentional reset event (AR), presentation of 4 dots for 33 ms. Subsequently, a contrast change (33 ms) occurred after 0.3–1.2 s, either within the same object that was cued or within the object on the opposite side (with equal probability). In the unimodal auditory-attention condition (second row) participants also fixated on the screen, but instead of visual objects, they were presented with Brownian noise in both ears. Here, the AR was a salient click in one ear, and the target consisted of a faint beep either in the ipsilateral or the contralateral ear. In bimodal trials (third and fourth row), visual objects and Brownian noise were presented simultaneously. Visual and auditory AR events were always spatially congruent. The target appeared in only one modality (i.e., either a contrast change or a beep) and participants reported, via button press, whether the target appeared on the left or right. Participants’ performance was held constant at ~66% by increasing or decreasing target saliency based on their modality-specific performance over the last six trials

The experimental procedure is illustrated in Figure 1: Participants were seated in front of a computer screen (ViewSonic TD2420, distance ~70 cm). At the beginning of each trial a white dot (1° visual angle), which the participants were required to fixate throughout the trial, appeared in the middle of a grey screen (RGB: 127, 127, 127). In the unimodal visual condition, after a variable time from 1 s to 1.2 s relative to trial onset, two circular drifting gratings appeared to the left and right of the fixation dot, respectively (please see supporting information of Landau & Fries, 2012, for detailed information about stimulus specifications). After another 1.25 s to 2.5 s, attention was reset towards one of the objects by four dots that briefly (33 ms) flashed around the respective object. Importantly, this attentional reset event (AR) provided no information about the location at which the target would appear. Subsequently, in a time interval between 0.3 s and 1.2 s after the AR (in increments of 16.6 ms, as predetermined by the refresh rate of the monitor), the target appeared in one of the two objects. The target itself consisted of a brief decrement in luminance within a circular patch in the centre of the respective grating. The participants’ task was to report, via button press (left or right), in which of the two objects the target had appeared. After the response, a colour change of the screen indicated, whether the participant’s answer was correct (green) or incorrect (red). If reaction time exceeded 1 s, the trial was classified as incorrect and the next trial started. In order prevent participants from guessing the target location, we instructed them to only respond when they actually perceived the target, and as a control, one fifth of the trials were catch trials in which there was no target present.

In the unimodal auditory attention condition, the temporal sequence of events and the response modalities were identical to the ones in unimodal visual. However, instead of two visual objects appearing on the screen, we presented 1/f^2^-noise (a.k.a. Brownian or red noise) to both ears of the participant. Following the approach of Ho et al. (2017), the noise streams played to the left and the right ear were time-reversed versions of each other and thus uncorrelated. The subsequent AR was a salient click in one ear, and the subsequent target was a faint beep (500 Hz; duration: 30 ms) in either the ipsilateral or the contralateral ear.

In the bimodal attention conditions, we distinguished between trials with visual targets and auditory targets. Stimulation during bimodal attention corresponded to the simultaneous presentation of the visual and auditory stimuli we used in the unimodal attention conditions. All the events before target presentation (i.e., object/noise onset, visual, and auditory AR) were temporally and spatially congruent, since an additional dissociation between congruent and incongruent ARs would substantially multiply the number of comparisons in our analysis (spatially congruent vs. incongruent; temporally congruent vs. incongruent; left/right vs. right/left; possible lateralization effects in the EEG, etc.), thus severely reducing statistical power. Only one target was presented per trial (i.e., either visual or auditory), and like in the unimodal attention conditions, our participants’ task was to report the target location (left/right), irrespective of the target modality.

Before starting the actual experiment, we asked each participant to perform a number of test trials in the bimodal attention conditions until their performance stabilized at around 66% correct in both, the visual and auditory modality. Subsequently, we used the respective contrast and loudness levels as initial stimulus parameters when we started the first block. In order to keep our participants’ performance level constant at around 66%, the stimulus intensity of the target (i.e., luminance contrast or loudness) was adaptively adjusted based on the participant’s modality specific performance in the last six trials. We monitored participants’ gaze via a remote video-based eye tracker (Eyelink 1000, SR research, Ontario, Canada). Whenever we detected eye movements towards one of the stimuli, we reminded the participants to not direct their gaze away from the fixation point. After a couple of test trials, none of the participants had problems maintaining fixation, so we did not analyse their eye movements in more detail.

### 2.3 | Behavioural data processing

In total, there were 55 different time points (between 0.3 s and 1.2 s relative to the AR) at which the target could appear. Within each condition, we presented the target four times per time point, that is two times ipsilateral and two times contralateral to the AR. For each participant and condition, the two responses per side and time point (1 = correct response, 0 = false response/miss) were averaged. This resulted in eight performance traces per participant (4 conditions × 2 locations with respect to the AR), which were subsequently smoothened by means of a 50-ms sliding window (cf. Fiebelkorn et al., 2013). Next, in order to remove possible confounds that might arise from our adaptive procedure, we performed logistic regression to estimate the variance explained by stimulus intensity. For further analysis we only kept the residuals, thereby eliminating the influence of stimulus intensity on behavioural performance. We computed the frequency spectra for the different conditions after zero padding the performance traces to a length of 4 s, thus obtaining a frequency resolution of 0.25 Hz. Note that due to the low-pass filter properties of the 50-ms sliding window, we subsequently only analysed frequencies from 2 to 10 Hz.

### 2.4 | EEG recording and pre-processing

EEG data were recorded using a 64-channel active electrode system (ActiChamp, Brain Products GmbH, Gilching, Germany) at a sampling rate of 1000 Hz. The electrodes were placed on the scalp according to the standardized international 10–10 electrode placement system, with the reference electrode being located over the left mastoid.

For preprocessing, we imported the data into EEGLAB (Delorme & Makeig, 2004) and removed line noise using Cleanline (https://www.nitrc.org/projects/cleanline/). Subsequently, we re-referenced the EEG to the average activity of all electrodes, filtered the data between 0.5 Hz and 100 Hz, down sampled it to 250 Hz, and cut it into epochs ranging from −1 s to 2 s relative to the attention reset event (AR). After visual inspection, trials and channels containing high amplitude noise and other easily identifiable confounds such as sudden electrode drifts and jumps were discarded. We performed independent component analysis (ICA) to decompose each participant’s data into maximally statistically independent signal sources (ICs). Next, we estimated the equivalent dipole location of each IC within an MNI (Montreal Neurological Institute) standard brain using the DIPFIT function as implemented in EEGLAB. Based on location, spectral properties and/or response to stimulation, we classified each IC to either reflect neural or non-neural activity. For further analysis, we only kept those ICs that we considered to be brain-related (i.e., ~17 ICs per subject, mean: 17.6, median: 16.5, range: 7 to 29). Subsequently, we back-projected the retained ICs to sensor space and interpolated the missing channels. Finally, we computed scalp current density (SCD, also called surface Laplacian) at each electrode, using FieldTrip, an open-source analysis toolbox for electrophysiological data (Oostenveld et al., 2011). As compared with the scalp potential, SCD emphasizes local activity of mainly cortical generators, while being relatively insensitive to more global activity and deeper sources (Pernier et al., 1988).

### 2.5 | EEG data analysis

After preprocessing, the EEG data were further analysed in FieldTrip. More specifically, we analysed the SCD at each electrode with respect to two time points, namely, the attentional reset event (AR) and target onset.

For the analysis with respect to AR, we baseline corrected each trial to the interval between −0.3 s and 0 s relative to AR onset. Subsequently, we computed event-related potentials (ERPs) and investigated the time course of spectral power in the interval between −0.3 s and 0.45 s relative to the AR. To this end, we segmented each trial into 1-s-long overlapping data windows advancing in 20-ms steps. Next, we multiplied each data segment with a Hanning window, padded it to a length of 4 s and computed the frequency spectra between 2 Hz and 12 Hz. Following this procedure, we obtained time-frequency representations (TFRs) with a frequency resolution of 0.25 Hz and a temporal resolution of 20 ms (Note, however, that due to the window length before padding the effective temporal and spectral resolution of each time-frequency-bin is limited to 1 s and 1 Hz, respectively). Averaging over trials yielded one TFR for each subject and experimental condition. Finally, we divided each TFR by the average power of each frequency during the baseline interval between −0.25 s and 0 s relative to the AR. Accordingly, all TFRs are expressed in units of relative change with respect to baseline.

In order to extract the alpha envelope of each trial, we first recalculated the time-frequency spectra between 9 and 11 Hz. To this end, we followed exactly the same procedure as described above, with the only exception that this time we used shorter data segments (0.25 s instead of 1 s) to achieve a better temporal resolution and thus retain larger portions of the data. In a next step, we averaged across frequencies to obtain a single time course representing the alpha envelope of the respective trial. Then, for each envelope, we computed the TFR between 2 and 10 Hz using 0.5 s data segments. Other than that, we employed the same parameters and procedures as before.

To investigate possible EEG signatures predicting detection performance, we analysed the data from −1 s to 0 s with respect to target onset. Note that while we included the very moment of target presentation in our analysis, we did not use any EEG data sample after that. Because it takes a visual stimulus at least 20–40 ms to reach the brain (cf. Jain et al., 2015), our analysis window lies well before the time at which any information about the visual stimulus enters the brain. For this reason, differences in the intensity of the target stimulus have no impact on our results and we can rule out any target onset effects in our spectral analyses. All parameters and procedures were the same as the ones we used for analysing the data with respect to the AR, except that we used shorter data segments (0.5 s) for time-frequency analysis. Note, that due to the window lengths we used for alpha envelope extraction (0.25 s) and the subsequent time-frequency analysis (0.5 s), the TFRs of alpha envelopes can only be represented up to 0.375 s before target onset (cf. Figure 6). Again, the respective TFRs are expressed in units of relative change with respect to baseline, which in this case ranged from −0.6 s to −0.5 s relative to target onset.

### 2.6 | Statistical analyses

For behavioural analyses, we used non-parametric permutation tests to assess the statistical significance of the observed effects (unless indicated otherwise in the Results). More specifically, we randomly shuffled the time points of the observed performance traces in each condition and for each participant, before applying the data processing steps described above (Fiebelkorn et al., 2013). Subsequently we repeated the analysis of interest. By reiterating these steps 1000 times, we obtained a distribution under the null-hypothesis against which we tested our observations.

Additionally, we used the free statistics software Jamovi to compare behavioural performance between conditions by means of a repeated-measures Bayesian ANOVA.

For the EEG analysis, statistical significance was computed by means of a cluster-based non-parametric permutation test (Maris & Oostenveld, 2007): Differences that exceeded a predefined threshold (two standard deviations from the mean in this case) are clustered and summed across adjacent channels, time points and frequency bins. By randomly exchanging conditions in a random subset of subjects before averaging and clustering, an alternative observation is obtained. Repeating this procedure multiple times (here: *n* = 1000) yields a reference distribution under the null hypothesis, which is then used to test how often clusters of the observed size are expected to randomly occur, while at the same time accounting for multiple comparisons over time samples and/or time-frequency bins.

## 3 | RESULTS

### 3.1 | Detection performance

First, we analysed detection performance collapsed over all conditions. If attention indeed operates on a supramodal level, the hypothesized ~4 Hz rhythm should dominate detection performance, irrespective of attention condition (unimodal/bimodal) or target modality (visual/auditory).

**FIGURE 2:**
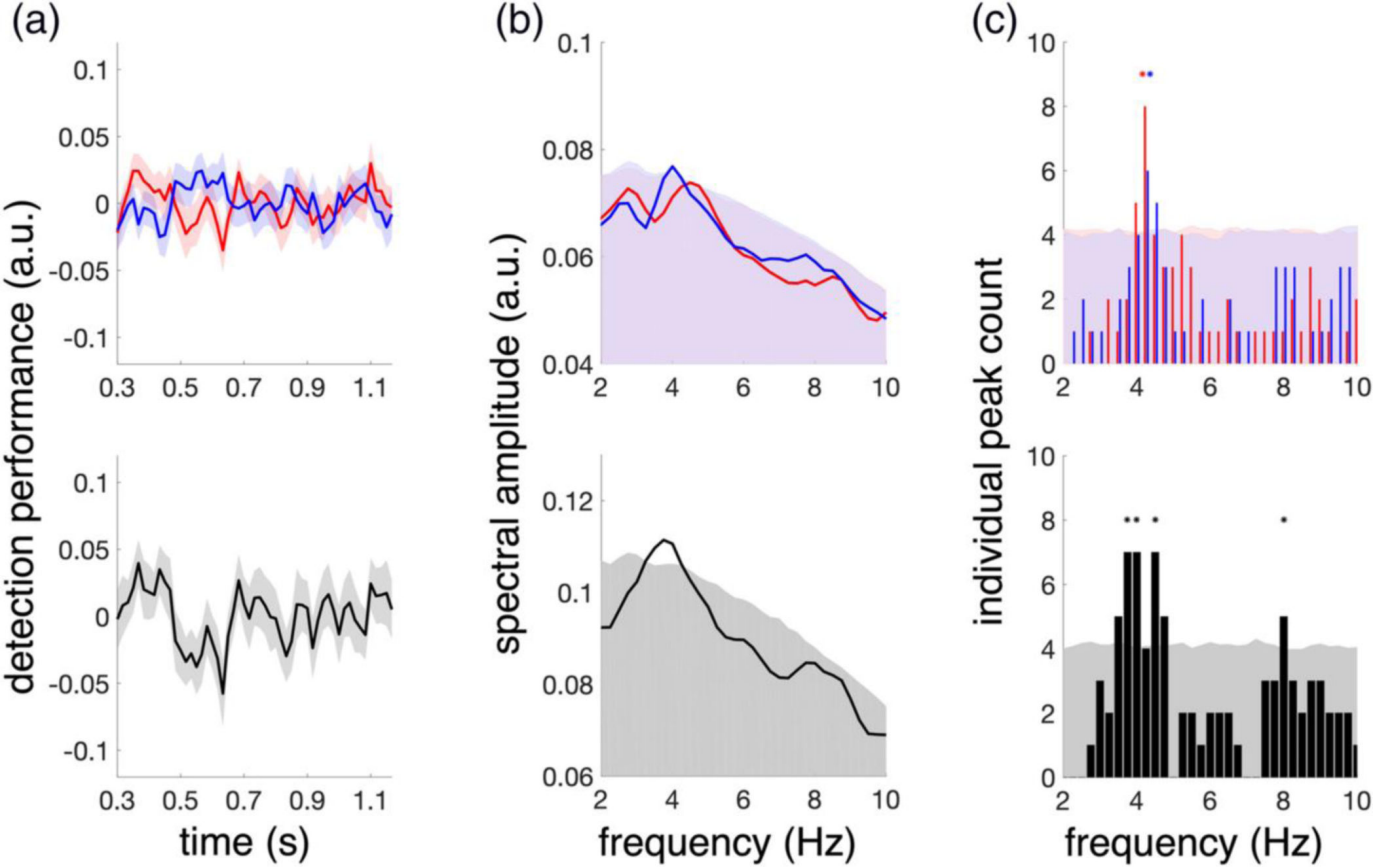
Detection performance across all conditions (visual, auditory, unimodal and bimodal). (a) Top: Average detection performance over time for targets ipsilateral (red) and contralateral (blue) to the attention reset event (AR). Bottom: Difference between ipsilateral and contralateral target detection traces (black). (b) Average spectra of detection performance traces. Note that the spectra were computed separately for each participant and condition before averaging and thus do not directly correspond to the performance traces depicted in (a). The shaded red, blue (overlapping regions in purple) and grey areas show the respective 95% confidence limits (uncorrected). (c) Counts of significant peaks in the spectra of individual subjects. Again, the red, blue, and grey shaded areas indicate the uncorrected 95% confidence limits and asterisks mark significant frequencies after correcting for multiple comparisons

For analysis in the time domain, we averaged detection performance traces over all conditions and participants, but separately for targets ipsilateral and targets contralateral to the attention reset event (AR). An examination of these averaged, behavioural time-series data suggest an initial inverse relationship between detection performance in response to the ipsilateral and contralateral targets (Figure 2a, top panel), with peaks in performance contralateral to the AR co-occurring with troughs in performance ipsilateral to the AR (and vice versa). The subtraction of these two conditions (i.e., ipsilateral–contralateral) emphasizes this initial counter-phase relationship (Figure 2a, bottom panel). However, these apparent behavioural oscillations are absent at longer cue-target delays. These averaged traces therefore suggest that the phase of attention-related rhythms in detection performance may lack consistency across participants, attention condition (unimodal/bimodal), and/or target modality (visual/auditory) (also see Figure S1).

Next, to analyse possible oscillations of detection performance traces irrespective of participant-level differences in phase, we computed each participant’s spectrum for ipsilateral and contralateral targets. Subsequently, we averaged the resulting spectra across participants. As shown in Figure 2b (top panel), the average spectra of both, ipsilateral and contralateral target detection traces, display ~4 Hz peaks, which exceed the 95% confidence level (not corrected for multiple comparisons). Applying the same procedure to the respective difference time courses (i.e., ipsilateral–contralateral) revealed substantial phase opposition at ~4 Hz (Figure 2b, bottom panel). Qualitatively, this finding is also supported by a direct comparison between phase angles of ipsilateral and contralateral detection traces, which, however, did not reach statistical significance (Figure S1).

Finally, we were interested whether significant 4-Hz oscillations could also be observed at the level of single participants. Therefore, we determined significant peaks in the spectrum of each individual. More specifically, we counted all participants that displayed spectral peaks that exceeded their individual 95% confidence level at each frequency (2–10 Hz). The results are shown in Figure 2c (top panel), where bars represent the number of individuals with significant peaks at each frequency and shaded areas indicate the number of expected peaks under the null hypothesis (i.e., the uncorrected 95% confidence limit over all participants). Indeed, a significant portion of participants displayed 4 Hz peaks in both the ipsilateral and contralateral target conditions as indicated by the asterisks in Figure 2c (top panel; FDR corrected for multiple comparisons). Peaks around 4 Hz were also significantly present in the difference spectra, as were peaks at 8 Hz (Figure 2c, bottom panel).

Thus far, our results are consistent with previous findings during unimodal visual attention (Fiebelkorn et al., 2013, 2018; Landau & Fries, 2012). More specifically, they confirm that objects presented ipsilaterally and contralaterally to the AR are sampled at frequencies around 4 Hz and in counter-phase (i.e., sequentially). Surprisingly, significant counter-phasic activity was also observed at 8 Hz, a frequency which previously has been related to attentional sampling within a single object, rather than two separate objects (Fiebelkorn et al., 2013) and which had not reached statistical significance in the average frequency spectra (i.e., Figure 2b).

**FIGURE 3:**
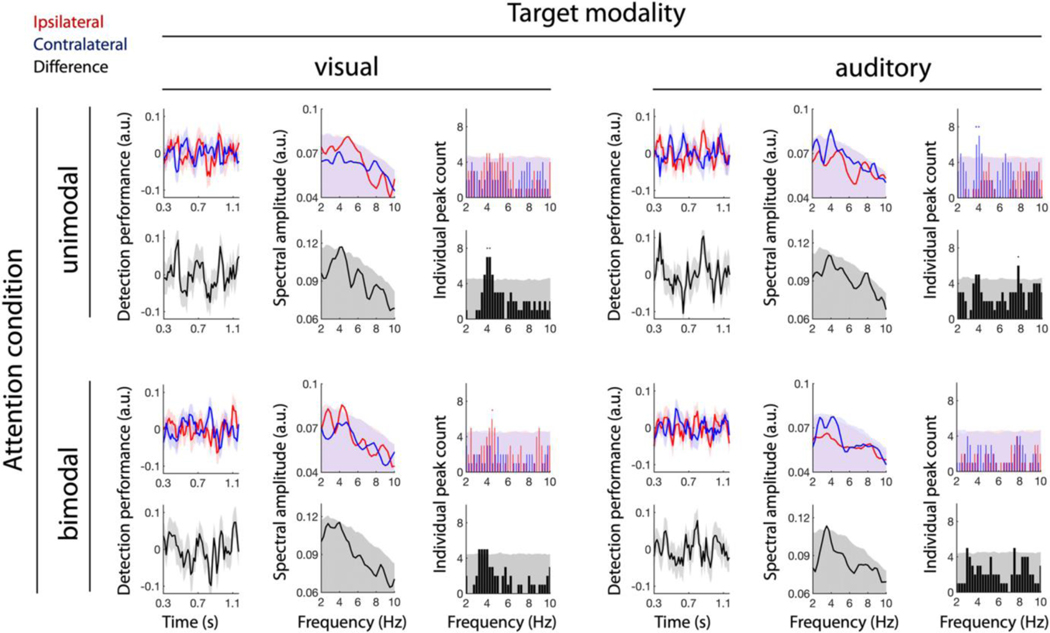
Detection performance by target modality and attention condition. Same conventions as in Figure 2. Detection performance traces, spectra, and individual peak counts are shown with respect to visual (left) and auditory (right) targets and for the unimodal (top) and bimodal (bottom) attention conditions

Next, we investigated how target modality and attention condition influence the observed attentional rhythms. Therefore, we repeated our analysis but this time separately for the different experimental conditions, that is, both visual and auditory target detection, as well as unimodal and bimodal attention conditions. The results are illustrated in Figure 3. Particularly in the unimodal attention conditions, the averaged, behavioural time-series data for both ipsilateral and contralateral targets (red and blue) appear to be more oscillatory and counter phasic than the ones we observed earlier in the averages over all attention conditions and target modalities (Figure 2a). This is also reflected in the difference traces (black). In the bimodal attention conditions, however, these oscillatory patterns are less pronounced in the averaged data.

In the frequency domain, after averaging the frequency spectra (FFTs) of the participant-level data, all four conditions displayed prominent spectral peaks around 4 Hz. However, the spectra and individual peak counts of ipsilateral and contralateral target-detection traces yielded a more ambiguous pattern than the corresponding averages over all conditions (Figure 2), which might partially result from the lower number of trials in the data. While in the visual modality ipsilateral target detection displayed stronger spectral modulations than contralateral target detection, the opposite held for the auditory modality. Moreover, peak frequencies of both, ipsilateral and contralateral detection spectra varied across attention and modality conditions.

In the difference spectra, by contrast, dominant ~4-Hz peaks are present in all four conditions, exceeding the 95% confidence limit. Additionally, we observed an 8-Hz peak in the unimodal auditory target detection task. These observations are also reflected in individual peak counts, where 4 Hz crossed the 95% confidence limit in all but the bimodal auditory condition, while 8 Hz became significant in the unimodal auditory condition only.

After correcting for multiple comparisons, only the 4-Hz peak in the unimodal visual and the 8-Hz peak in the unimodal auditory condition remained significant (as indicated by asterisks in Figure 3). Again, these findings are qualitatively supported by the phase angle differences between ipsilateral and contralateral detection traces (Figure S2).

Overall, these results do not yet answer our primary question, namely, whether sampling frequency or phase opposition between objects changes depending on attention condition and/or target modality. Thus, in order to investigate whether the difference spectra were similar or differed across conditions we calculated a Bayesian repeated-measures ANOVA for frequencies from 3.5 to 4.5 Hz. The analysis yielded Bayes factors (BF_10_) of ~0.3 and less for any effect across conditions (attention: BF_10_ = 0.196; modality: BF_10_ = 0.310, attention*modality: BF_10_ = 0.245, see Tables S1 and S2 for more details), thus providing substantial evidence for the null hypothesis, namely, that 4-Hz phase opposition between ipsilateral and contralateral target detection does not depend on attention condition or target modality.

In sum, the unexpected peak at 8 Hz might indicate that the presented auditory streams are possibly perceived as a single object with different target locations, rather than two separate objects (see Section 4).

Most importantly, the 4-Hz and 8-Hz effects observed in the overall performance (Figure 2) could only be verified in the unimodal visual and auditory conditions (Figure 3). However, a Bayesian repeated-measures ANOVA provided evidence that ~4-Hz phase opposition does not significantly differ across conditions. This argues in favour of the idea that attentional 4-Hz rhythms operate at a supramodal level.

### 3.2 | Neural responses to attentional resets

If attentional shifting between objects or locations is associated with theta oscillations, one would predict evoked theta responses, or the phase reset of ongoing theta oscillations due to the AR in either modality. Moreover, responses to left and right ARs should display phase opposition in the theta range.

**FIGURE 4:**
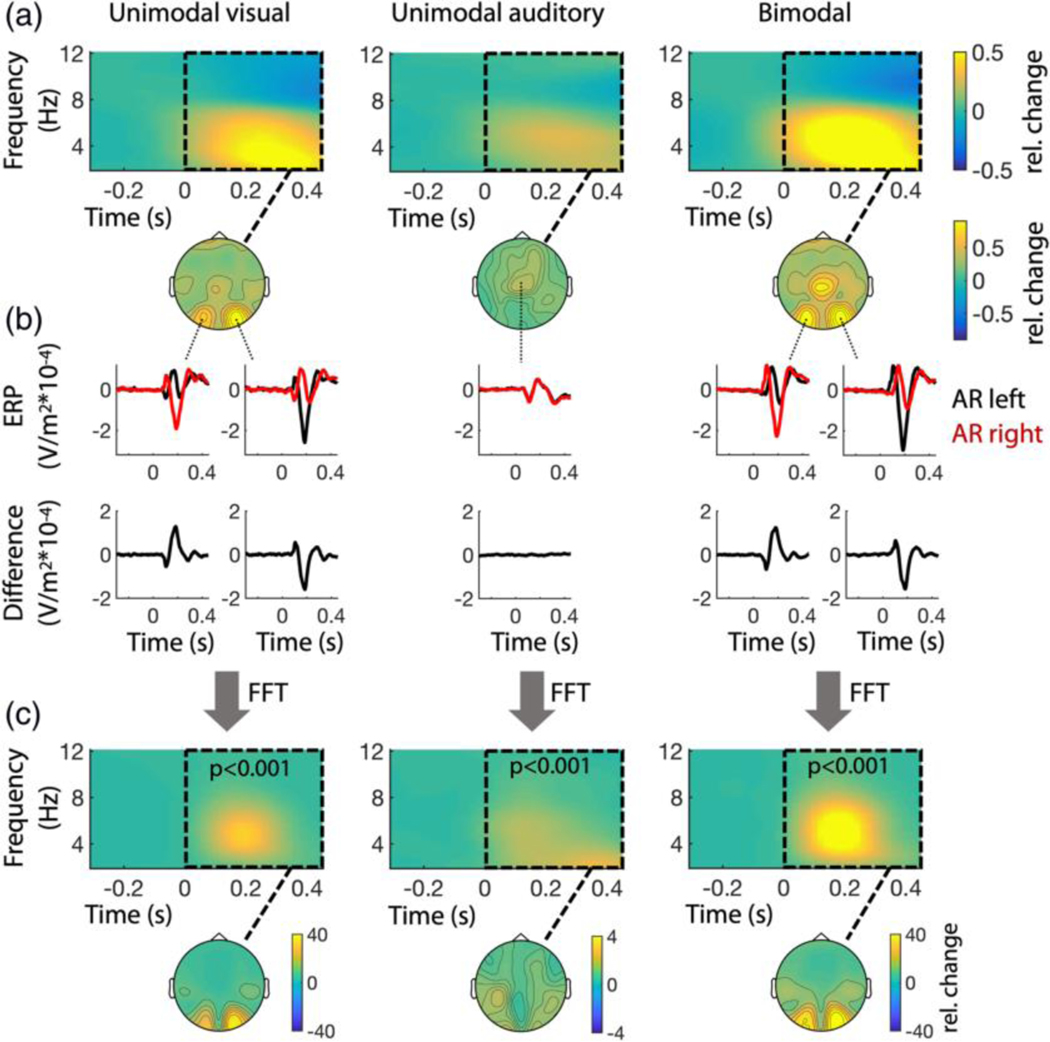
Neural responses to attentional reset (AR) events. Time 0 indicates the time of the AR. (a) Time-frequency analysis with respect to visual, auditory, and bimodal ARs, averaged over left and right ARs and over all channels. The topographic patterns of the respective responses (time and frequency range are indicated by the dashed boxes) are shown below. (b) ERPs at those channels that displayed the most prominent power increases (over left visual cortex: P3, P5, P7, PO3, PO7, O1; over right visual cortex: P4, P6, P8, PO4, PO8, O2; centrally: FC1, FCz, FC2, C1, Cz, C2). The first row shows ERP responses to left (black) and right (red) ARs. The respective differences in the second row (black) serve as an indicator of phase opposition, that is, signal components in counter-phase are enhanced, while in-phase components cancel each other. (c) Time-frequency-analyses of the differences in (b) reveal significant phase opposition, which is most prominent in the theta-range. The topographies below show the respective spatial distribution across the scalp

The respective analyses (Figure 4) confirm these predictions: In the frequency domain, we observed significant power increases at frequencies between approximately 2 and 8 Hz. (Figure 4a, top panel). The corresponding topographies display patterns typical of stimulation in the visual and auditory modality (Figure 4a, bottom panel). At sites associated with visual and auditory responses, respectively, bimodal ARs evoked stronger responses than unimodal ones. This might be explained by the principle of inverse effectiveness, which states that the strength of multisensory integration increases, as the responsiveness to individual sensory stimuli decreases (in this case due to divided attention; see, e.g., Holmes, 2009).

Altogether, the observed effects in the frequency domain seem to primarily reflect the respective ERP responses shown in Figure 4b. Moreover, at occipital sites where visual stimulation evoked the strongest responses, ERPs following left and right ARs (Figure 4b, first row, depicted in black and red, respectively) display opposite phases. However, this only holds for visual ARs but not for auditory ones, presumably because responses to the latter are not lateralized at the scalp level. Following the same rationale as in the behavioural analysis before, we utilized the differences between ERPs evoked by left and right ARs (Figure 4b, second row) as an indicator of phase opposition. As expected from the ERPs above, larger differences are only observed at occipital channels in response to visual ARs.

To determine the frequencies with the strongest phase opposition, we employed a similar approach as in our behavioural analysis. More specifically, we performed a time-frequency analysis of the difference traces shown in Figure 4b (second row, black). The resulting spectral patterns (Figure 4c) differed from the ones we found in the respective power spectra averaged over left and right ARs (Figure 4a, lateralized responses are shown in Figure S3), in that they were more focal in both frequency (i.e., confined to the theta band) and time. To compare our approach to more classical measures of EEG phase, we additionally computed inter-trialcoherence (ITC) to quantify phase consistency over trials and the phase-opposition-sum (POS, cf. VanRullen, 2016a) as an alternative measure for counter-phasic activity (Figure S3). Overall, the results of our approach using difference spectra (Figure 4c) and the POS (Figure S3) are largely identical, especially regarding the scalp topographies. Note, however, that in the difference spectra the effects become even more prominent than in the POS and furthermore reveal significant phase opposition in the auditory condition.

Again, the observed patterns suggest that power, phase consistency and phase opposition in response to ARs can at least partially be dissociated based on their respective time and frequency range. Altogether these observations suggest that phase opposition in the theta range is not merely an epiphenomenon of stimulation induced power changes, but rather reflects a theta-rhythmic attentional oscillation between the two objects.

### 3.3 | Attentional resets modulate alpha envelope

**FIGURE 5:**
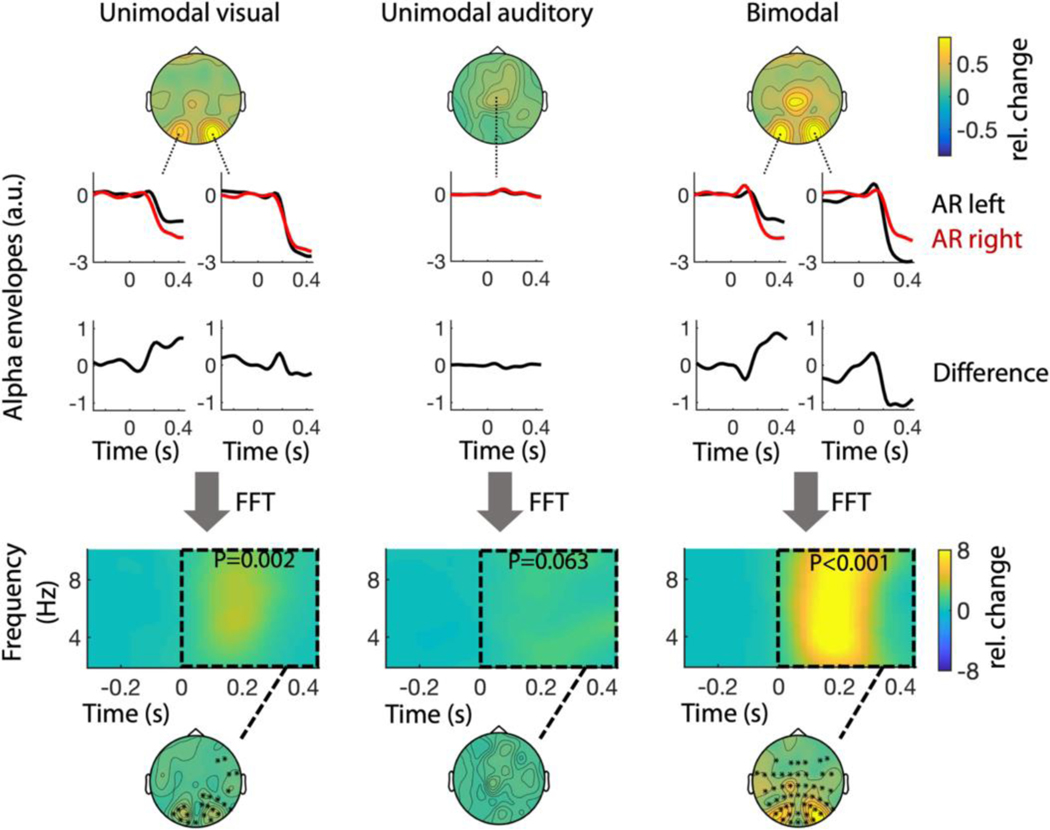
Modulation of alpha power in response to ARs. At sites that display strong sensory theta responses to the AR (top), the alpha power envelopes (middle) are modulated differently for left (red) and right (black) ARs. Time-frequency analyses of the respective differences (bottom) indicate that the alpha power envelopes fluctuate in counter-phase most prominently at frequencies in the theta-range. The strongest modulation differences (significant channels are marked with asterisks) largely coincide with the sensory theta responses

So far, we found that ARs induce theta-rhythmic modulations of both detection performance and EEG activity. Furthermore, there is evidence that rhythmic attentional switches between objects are also reflected in modulations of alpha power envelopes (Fiebelkorn et al., 2019; Jia et al., 2017). We hypothesized, that if these envelope modulations are directly related to the attentional rhythms, we observed earlier, they should occur within same frequency range (~4–8 Hz) and likewise display phase-opposition between left and right ARs. To investigate this, we examined the impact of attention resets (AR) on the alpha power envelope. To extract the envelopes, we first calculated the time-frequency spectra for frequencies between 9 and 12 Hz and subsequently averaged them into a single time course. Figure 5 shows the resulting alpha envelopes corresponding to left (red) and right (black) ARs at electrode sites that displayed the strongest sensory responses to the AR.

To investigate whether alpha envelopes following left and right ARs oscillate in counter phase, we evaluated phase opposition in the same way we did before in our analyses of behavioural performance and sensory responses. More specifically, we took the differences between left-AR and right-AR envelopes (Figure 5) and analysed their spectral content by means of a time-frequency analysis. The difference spectra again show that phase opposition of alpha envelopes is most concentrated in the theta band, although this only becomes significant in the unimodal visual and in the bimodal condition, respectively. Consistent with our earlier observations (Figure 4) the neural responses were stronger in the bimodal condition.

### 3.4 | Alpha envelope modulation predicts detection performance

Thus far, we have demonstrated AR-induced theta-phase opposition in both behaviour and EEG activity. However, in order to establish a direct link between behavioural and neural attentional rhythms we would have to demonstrate that counter-phasic neural theta activity is predictive of detection performance. To this end, we computed the target-locked time-frequency spectra and compared the power and phase between hit and miss trials. However, we did not find any significant effects in the theta range itself (see Figure S4). Therefore, we hypothesized that behavioural performance might instead be associated with theta modulated alpha activity.

**FIGURE 6:**
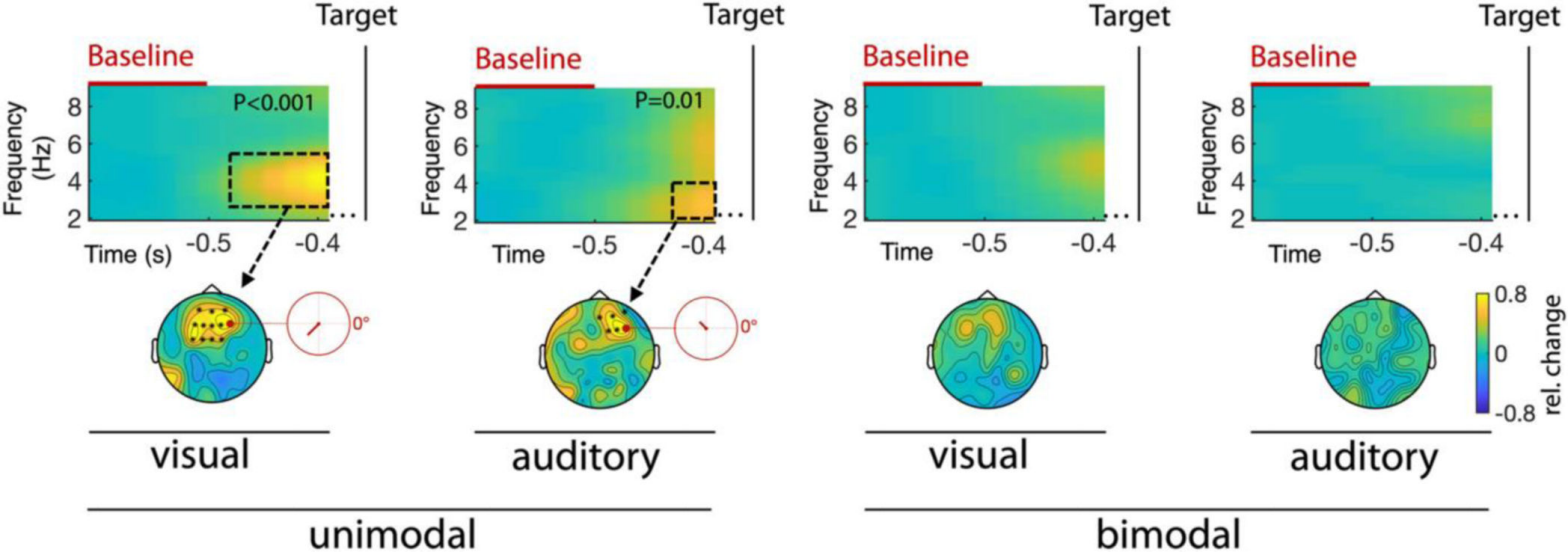
Alpha envelopes of hit versus miss trials. Time is displayed relative to target onset. The missing interval between −0.4 s and target presentation is due to the length of our analysis windows for both, the extraction of the alpha envelopes and the time frequency transformation of the latter. The relative power changes with respect to the baseline period (<−0.5 s) serve as a measure for counter phase between alpha envelopes related to hit and miss trials respectively. The dashed boxes encompass the time-frequency ranges displaying significant effects and the asterisks in the topographies demark significant channels. The red circles in the unimodal conditions show the respective angular distances (i.e., hit- vs. miss-trial envelopes) at those frequencies and electrodes that displayed the strongest effect (visual: 4 Hz at electrode F4; auditory: 3 Hz at electrode F6)

To investigate this possibility, we extracted alpha envelopes and determined the degree of phase opposition using the same strategy we employed in the analyses before. For each participant, we subtracted the alpha envelope preceding miss trials from the one preceding hit trials. Subsequently we performed a time-frequency analysis on the resulting differences. Note, that we only used the data portion before target onset so that our analysis would not contain any neural responses to the target stimulus. As shown in Figure 6, in the unimodal conditions the alpha envelopes of hit and miss trials were modulated in counter-phase at ~4 Hz (visual) and <4 Hz (auditory). Remarkably, in both conditions, the respective phase opposition was strongest at frontal channels and not in early sensory areas, where we observed the strongest envelope modulations in response to the AR. In the bimodal conditions these effects were reduced and did not reach statistical significance. Overall, these findings are consistent with our behavioural results.

## 4 | DISCUSSION

Extending an established visual paradigm (Landau & Fries, 2012) into a combined auditory–visual one, we confirmed earlier findings of rhythmic attentional fluctuations in the theta range (4–8 Hz). More specifically, we demonstrated that visual-detection performance alternates between objects at ~4 Hz. This is generally interpreted as evidence that visual attention samples two simultaneously presented objects in sequence (Fiebelkorn et al., 2013; Jia et al., 2017; Landau & Fries, 2012).

However, while visual attentional theta rhythms have been shown in a number of studies (Fiebelkorn et al., 2013, 2018; Helfrich et al., 2018; Huang et al., 2015; Landau et al., 2015; Landau & Fries, 2012), reports of similar effects in the auditory modality have been rare and inconsistent (Ho et al., 2017; Ilhan & VanRullen, 2012; Ng et al., 2012; Zoefel & Heil, 2013). Ilhan and VanRullen (2012) for instance were not able to find the rhythmic modulations of attention-related neural activity, which they had previously demonstrated in the visual modality (VanRullen & Macdonald, 2012). By contrast, Ng et al. (2012), found that auditory stimulus detection is —at least partly—contingent on the power and phase of ongoing neural theta oscillations. However, a study by Zoefel and Heil (2013) suggests that correlations between auditory stimulus detection and the phase of low-frequency oscillations may be artefacts, resulting from amplitude differences between the respective event-related potentials. Still, such possible artefacts are not able to explain the rhythmic modulations of behavioural detection performance, as they have recently been demonstrated by Ho et al. (2017) and Kayser (2019), for instance. One likely reason for the discrepancies between the aforementioned studies is that they used paradigms and methods that differed considerably not only among each other but also from the ones employed in the visual domain. By contrast, here we measured auditory-detection performance in a task that closely corresponded to the visual paradigm, thereby permitting a direct comparison of the results in the two modalities for the first time.

In our paradigm, the temporal pattern of auditory-detection performance displayed significant phase opposition at ~4 Hz and at 8 Hz, but only the effect at 8 Hz in the unimodal condition remained significant after correcting for multiple comparisons. The observation of 8-Hz phase opposition was unexpected, since we anticipated the hypothesized supramodal attentional mechanism to oscillate between two objects at a rate of ~4 Hz – irrespective of modality. With regard to the significant peak at 8 Hz, it has been demonstrated that attention samples target locations within a single object at ~8 Hz (Fiebelkorn et al., 2013; Gaillard et al., 2020; VanRullen & Macdonald, 2012).

Thus, our results may indicate that the two presented auditory noise streams are not consistently perceived as two independent objects (as we had initially reasoned), but instead may be perceptually merged into a single object with two target locations. In their study, from which we adapted our auditory stimuli, Ho et al. (2017) argue, that their ‘left- and right-ear maskers were time-reversed versions of each other such that they were clearly lateralized and uncorrelated’. However, the effects they find also display oscillations at ~8 Hz rather than 4 Hz, which might indicate that in their experiment, too, the two physically independent streams were actually perceived as one. Either way, our results confirm that attentional rhythms are not exclusively occurring in the visual modality but also occur in the auditory modality, at least during unimodal attention.

Based on the proposal that attention samples single objects at 8 Hz and two simultaneously presented objects sequentially at 4 Hz, we would expect the attentional rhythm in the bimodal attention conditions to further divide across objects and thus to shift towards frequencies around 2–3 Hz (i.e., 8 divided by 3 or 4, depending on whether the subject perceived the two auditory streams as a single object; Holcombe & Chen, 2013). However, although the effects observed in the unimodal conditions were reduced and did not remain significant after correcting for multiple comparisons, the strongest phase opposition was still observed at frequencies around 4 Hz.

Moreover, the low Bayes Factor speaks in favour of the null hypothesis, that is, a true absence of such an effect (although a possible lack of statistical power cannot be ruled out), rather than an actual difference in 4-Hz phase opposition among the four conditions. One explanation for this observation, namely, that the sampling frequency is not further reduced in the bimodal conditions, might be that the presented visual and auditory stimuli are integrated into two multisensory objects.

Another possibility is that 4 Hz constitutes the basic attentional sampling frequency across objects, irrespective of their number. From this point of view, the reduction of effects in the bimodal attention conditions may be explained by an irregular sampling sequence between the objects (i.e., the order in which the objects are sampled changes). This interpretation is consistent with a recent proposal that sensory and motor aspects of environmental sampling are organized by a shared theta rhythm in the attention network (Fiebelkorn & Kastner, 2019). In addition to previous evidence that attention-related sampling occurs at a low-theta frequency (Fiebelkorn et al., 2018; Helfrich et al., 2018), the likelihood of exploratory eye movements also occurs at a low-theta frequency (Bosman et al., 2009; Otero-Millan et al., 2008).

In line with our behavioural observations, our EEG results imply a reset of attention causes theta-rhythmic neural activity to occur counter-phasically between two attended objects/locations. Moreover, in the same respective occipital channels, this theta activity also appeared to modulate the magnitude of alpha power. Increased alpha power in sensory cortex has often been associated with an active suppression of sensory processing (Foxe & Snyder, 2011). As such, the present results are therefore not only consistent with a theta-rhythmic enhancement of visual processing alternating between the target objects/locations but also with a theta-rhythmic suppression of visual processing alternating between the target objects/locations (Fiebelkorn & Kastner, 2019). That is, periods of relative sensory enhancements at one object/location are associated with periods of relative sensory suppression at the other object/location. Moreover, the spectral patterns in both our measure of phase-opposition (Figure 4c) and the phase-opposition-sum (POS, Figure S3) are quite different from the ones of stimulus-induced power or ITC increases (see Figures 4a and S3). More specifically, they are much more focal in both time and frequency (i.e., confined to the theta band). This strongly suggests that phase opposition in the theta band reflects an attentional process that cannot be explained by stimulation alone. Therefore, we consider the observed alpha modulations likely to reflect attentional switches between objects, rather than mere neural responses to sensory stimulation. Admittedly, in the alpha range (Figure 5), this does not become as clear, because the FFT is limited to the theta range (although here the spectra peak between 4 Hz and 8 Hz, too). However, it is difficult to conceive how the responses to the AR could result in theta modulated alpha envelope differences other than by attention, especially in light of the findings presented in Figure 4.

It should furthermore be noted that our approach to detect phase opposition, namely by subtracting the time series of two conditions and then computing the frequency spectrum, appeared to be more sensitive than other, more conventional methods, such as computing the angular distance between the respective time series or the POS (VanRullen, 2016a). One possible reason is that subtraction reduces unsystematic variance in the data and thus may render our approach more robust in the presence of noise. However, applying the aforementioned classical measures supported our conclusions at least on a qualitative level. Finally, while the results did not show a direct relationship between behavioural and neural oscillations (e.g., in the form of phase coherence), we did demonstrate that detection performance was contingent on theta-modulated frontal alpha (~8–12 Hz) activity. This is also in line with earlier studies that found frontal alpha to be the most predictive neural indicator for stimulus detection or discrimination performance (e.g., Busch et al., 2009; Strauß et al., 2015).

### 4.1 | Potential limitations

Overall, our data are consistent with our initial hypothesis that visual and auditory attentional rhythms are not independent but driven by an inter- or even supramodal mechanism. Note, however, that the number of presented and/or perceived objects changed across conditions: First of all, we presented two physically different stimuli in the unimodal and four stimuli in the bimodal conditions. Second, it is possible that the two auditory stimuli were perceived as one single stream of noise, rather than two separate objects. Third we do not know, if and how far visual and auditory stimuli were perceptually integrated to form multisensory objects. Therefore, our paradigm did not allow us to demonstrate the hypothesized supramodality of attention directly. Instead, our results raise questions, that we did not anticipate in our experimental design, as, for instance, if and in how far the attentional sampling frequency is affected by the number of presented objects. Additionally, the number of attention and target stimulus combinations may have resulted in a lack of power that prevented us from providing more clear-cut answers. Experiments with only two objects per trial (i.e., either two visual, two auditory or one visual and one auditory object) may thus help to shed further light on these questions.

## 5 | CONCLUSION

Leveraging a novel auditory–visual detection paradigm, the present results contribute to a growing body of evidence that attentional sampling is a fundamentally rhythmic process, not only in the visual modality, but across sensory modalities. As such, a supramodal attentional rhythm might provide the mechanistically required flexibility by which different sensory regimes and the motor system can be coupled and decoupled depending on the task at hand.

## Supplementary Material

Supplemental 1

## Data Availability

The data that support the findings of this study are available from the corresponding author, MP, upon reasonable request.

## Abbreviations:

AR: attentional reset event
EEG: electroencephalogram
ERP: event-related potential
FFT: frequency spectrum/(fast) Fourier transform
IC: independent component
ICA: independent component analysis
ITC: inter-trial-coherence
POS: phase-opposition-sum
SCD: scalp current density
TFR: time-frequency representation
