# Supplementary material for: Attentional sampling of visual and auditory objects is captured by theta-modulated neural activity
Source: Eur J Neurosci. Author manuscript; Available in PMC 2023 Jun 9. (PMC10251135; doi:10.1111/ejn.15514)
Supplement: Supplemental 1 [file NIHMS1891535-supplement-Supplemental_1.docx]

**Supplemental Material**

**
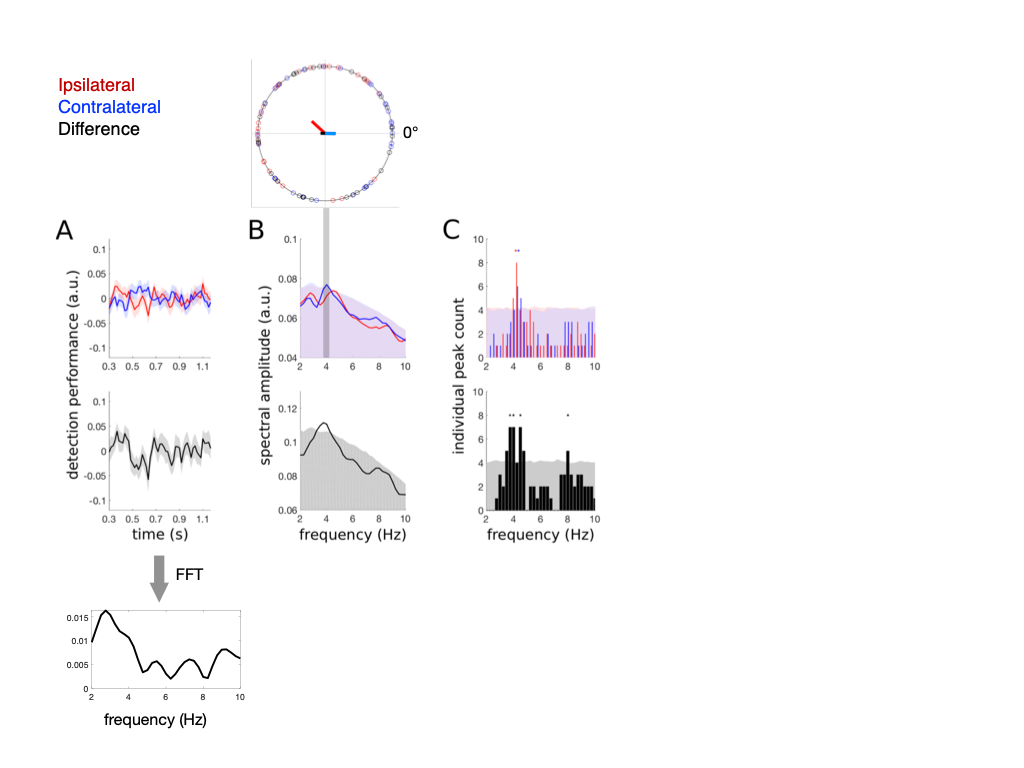
**

**Supplemental Figure 1: Additional analyses of phase consistency over all conditions.** Here we show an extended version of Figure 2. Below the black difference trace in (A), we additionally display the spectrum of the averaged time series. In contrast to the spectra in (B), which were computed for each participant separately before averaging, the peak frequency here is observed at 3 Hz instead of 4 Hz and its magnitude is almost ten times smaller. This supports our conclusion that detection performance is not constantly phase consistent across participants, attention condition (uni-/bimodal), and/or target modality (visual/auditory). In Figure 2B we found the strongest phase opposition at 4 Hz. In the polar plot above, we additionally display the 4 Hz phase angles for each participant (red = ipsilateral, blue = contralateral, black = angular distance). The short length of resulting average vectors again supports the notion, that phase may not be very consistent across participants and conditions. However, the vectors do display opposite phase angles between ipsi- and contralateral detection performance.


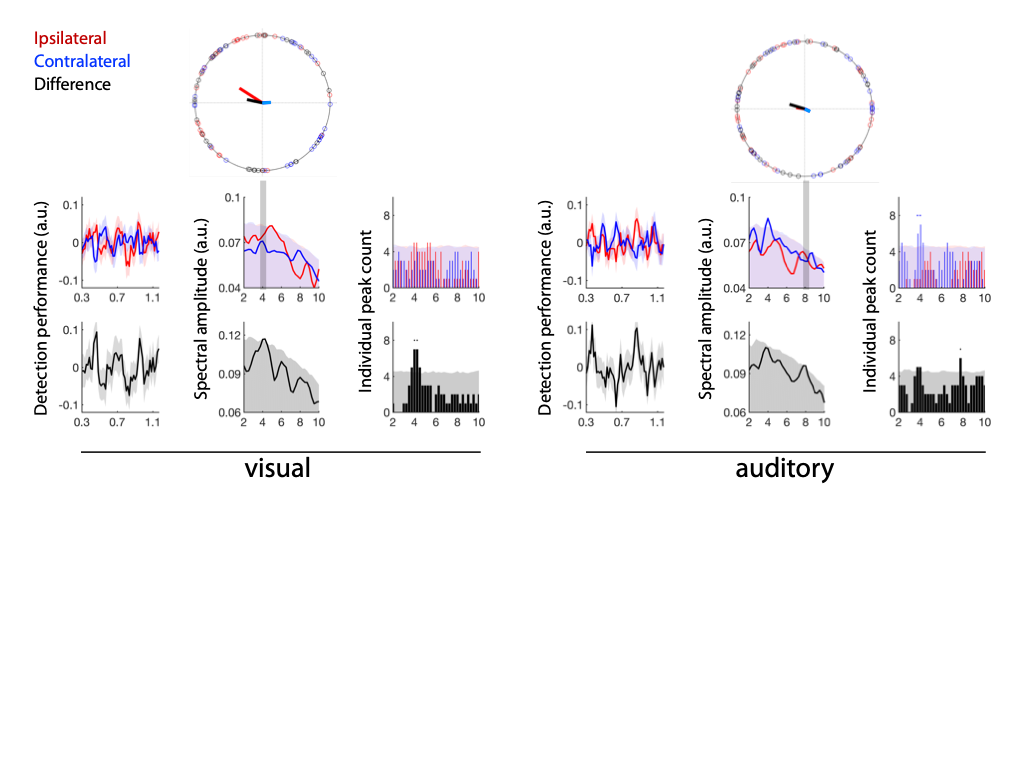


**Supplemental Figure 2: Phase angles in the unimodal visual and unimodal auditory attention conditions.** Again, we analyzed the phase angles of ipsi- and contralateral spectra at the frequencies, where we found significant phase opposition in the unimodal visual and unimodal auditory attention conditions (i.e., 4 Hz and 8 Hz respectively). Like in the overall spectrum (Supplemental Figure 1) the vector angles display opposite phases, thus supporting our findings in the difference spectra (black).

| **Models** | | **P(M)** | **P(M\|data)** | | | **BF _M_** | | | **BF _10_** | | | **error %** | | |  |
| --- | --- | --- | --- | --- | --- | --- | --- | --- | --- | --- | --- | --- | --- | --- | --- |
| Null model (incl. subject) |  | 0.200 |  | 0.630 |  | | 6.814 |  | | 1.000 |  | |  |  | |
| attention |  | 0.200 |  | 0.123 |  | | 0.563 |  | | 0.196 |  | | 1.931 |  | |
| modality |  | 0.200 |  | 0.195 |  | | 0.971 |  | | 0.310 |  | | 0.757 |  | |
| attention + modality |  | 0.200 |  | 0.041 |  | | 0.170 |  | | 0.065 |  | | 3.966 |  | |
| attention + modality + attention  ✻  modality |  | 0.200 |  | 0.010 |  | | 0.042 |  | | 0.016 |  | | 5.246 |  | |
|  | | | | | | | | | | | | | | |  |
| *Note.*  All models include subject. | | | | | | | | | | | | | | |  |

**Supplemental Table 1: Bayesian Repeated Measures ANOVA.** To investigate whether the difference spectra are similar or differ across conditions we calculated a Bayesian Repeated-Measures ANOVA for frequencies from 3.5 to 4.5 Hz. The table shows the comparison between models of increasing complexity (individual conditions, combined conditions and combined conditions including interaction) and the null model. The Bayes Factors (BF_10_) of all model comparisons are substantially lower than 1 for any effect across conditions. This is generally interpreted as substantial evidence for the null hypothesis. In our case that is 4 Hz phase opposition between ipsi- and contralateral target detection does not depend on attention condition or target modality.

| **Models** | | **P(M)** | | **P(M\|data)** | | **BF_M_** | | **BF_10_** | | **error %** | | |
| --- | --- | --- | --- | --- | --- | --- | --- | --- | --- | --- | --- | --- |
| Null model (incl. attention, modality, subject) |  | 0.500 |  | 0.803 |  | 4.084 |  | 1.000 |  |  |  |  |
| attention ✻ modality |  | 0.500 |  | 0.197 |  | 0.245 |  | 0.245 |  | 4.77 |  |  |
| *Note.* All models include attention, modality, subject. | | | | | | | | | | | | |
|  | | | | | | | | | | | | |

**Supplemental Table 2: Interaction only.** The table shows the Bayesian Repeated-Measures ANOVA but this time for the interaction model (attention*modality) only, i.e. the main effects of attention and modality are included in the null model. Again, the data provides evidence for the null hypothesis.


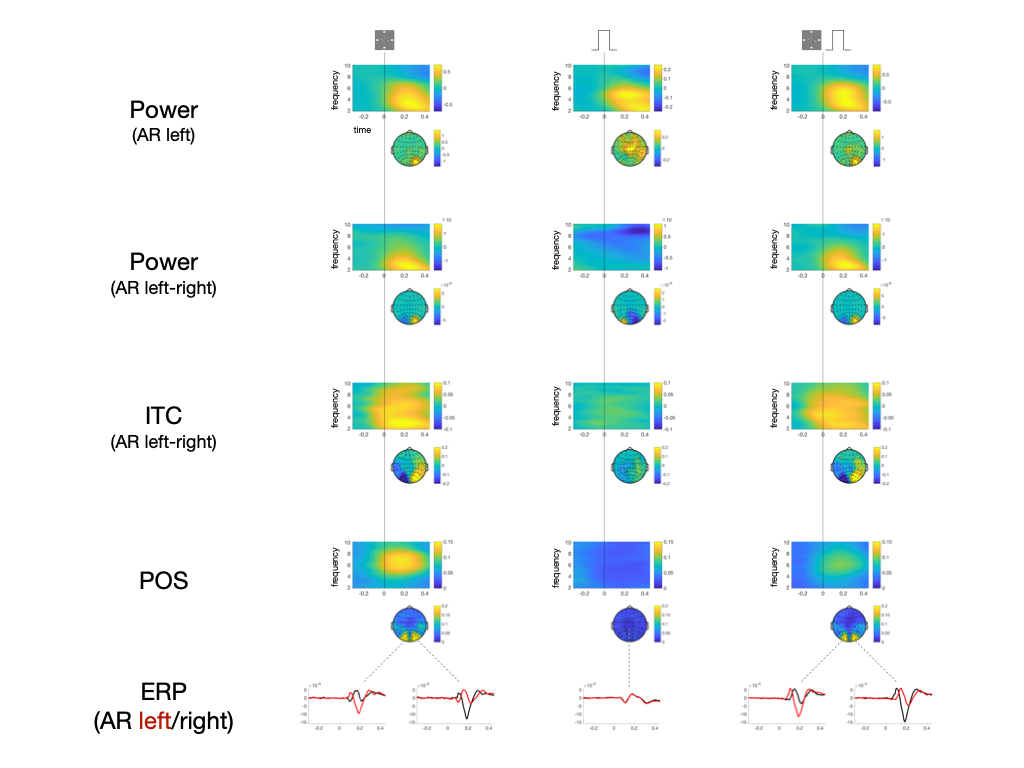


**Supplemental Figure 3: Additional power and phase effects in response to attentional resets (AR).** The first two rows show the power changes in response to left ARs and the power difference between responses to left and right ARs, respectively. The third row shows the inter-trial-coherence (ITC) as a measure of phase consistency across trials. The substantial increase in ITC suggests an AR induced phase reset. As the ITC response comprises a much broader frequency range than the respective power increase, this phase reset cannot be fully attributed to an ERP related power increase alone. In the third row, the phase-opposition-sum (POS = ITC_left_ARs_ + ITC_rightARs_ – 2 * ITC_allARs_, cf. VanRullen, 2016) indicates phase opposition between neural responses to left and right ARs, respectively. Note that the corresponding frequency range is much more focal than the ones for power and ITC and confined to the theta band. Moreover, both the time-frequency representations and the topographies are consistent with our findings based on the alternative approach shown in Figure 4C, which appears to be even more sensitive than the POS.

**Supplemental Figure 4: Performance-related spectral analysis relative to target onset**. In the frequency range below 12 Hz the comparison between hit and miss trials just before target onset did not yield any significant or otherwise noticeable effects in power (top row) or phase consistency across trials (ITC, second row). Likewise, we observed no phase opposition (POS) between hit and miss trials. Overall, these findings suggest, that theta power and phase are not directly indicative of detection performance.
